# Primary Care Physicians’ Interpretation of Smartwatch ECGs in Atrial Fibrillation: A Cross-Sectional Study

**DOI:** 10.1016/j.jacadv.2026.102855

**Published:** 2026-06-02

**Authors:** Yuval Avidan, Baruch Weizman, Margalit Vainerovsky, Sameha Zahra, Stav Brodsky, Mordechai Alperin, Jorge E. Schliamser, Avinoam Shiran, Michael Hauzer, Asaf Danon

**Affiliations:** a Department of Cardiology, Lady Davis Carmel Medical Center, Haifa, Israel; b Department of Internal Medicine, Lady Davis Carmel Medical Center, Haifa, Israel; c Department of Family Medicine, Meuhedet Health Services, Pardes Hana-Karkur, Israel; d Department of Family Medicine, Haifa & Western Galilee District, Clalit Health Services Community Division, Haifa, Israel; e Ruth and Bruce Rappaport Faculty of Medicine, Technion- Israel Institute of Technology, Haifa, Israel; f Department of Family Medicine, Faculty of Medicine, Technion-Israel Institute of Technology, Haifa, Israel

**Keywords:** atrial fibrillation, primary care physicians, smartwatch ECG

Smartwatches have evolved from timekeeping and fitness tracking to enabling on-demand electrocardiogram (ECG) recordings, with established validation for atrial fibrillation (AF) detection.^1^ Guidelines endorse their use for AF screening in selected populations,^1^ specifying that a definitive diagnosis requires review by “a physician with expertise in ECG rhythm interpretation”; however, what constitutes sufficient expertise remains undefined. As smartwatch adoption grows, primary care physicians (PCPs) are increasingly expected to interpret data from consumer-grade devices—a task for which many have limited training. Despite their central role in outpatient care, the ability of PCPs to interpret AF from smartwatch recordings remains uncharacterized. The aim of the study was to determine PCPs’ accuracy in identifying AF using smartwatch ECGs and the electrocardiographic factors influencing diagnostic performance.

## Methods

We conducted a questionnaire-based, cross-sectional study recruiting PCPs from diverse regions and health care settings in Israel during June 2025, following STROBE (Strengthening the Reporting of Observational Studies in Epidemiology) guidelines. Participants were invited through closed professional social media groups and direct peer contacts. The web-based questionnaire included 40 smartwatch ECGs: 10 true positive, 10 true negative, 10 false positive, and 10 false negative AF cases, as determined by the Apple Watch Series 6 automated interpretation and confirmed by 2 consultant cardiologists, with a third adjudicator. All ECGs were obtained from a prospective database of hospitalized adults. Only tracings interpreted as sinus rhythm (SR) or AF were included; uninterpretable recordings and atrial flutter cases were excluded. Intermittently paced rhythms were retained and labeled. To simulate real-world scenarios, the device’s automated interpretation was retained and a vignette describing a 70-year-old man with palpitations. The questionnaire comprised 2 randomized sets of questions: 20 cases labeled “Doctor, my watch indicated AF” (10 true positive and false positive), and 20 cases labeled “Doctor, I had palpitations, but my watch indicated SR” (10 true negative and false negative). Participants classified each ECG as AF, SR, another rhythm, or “I don’t know–I would consult a colleague.” A 30-minute time limit was set, no feedback was provided, and participants had no prior exposure to the tracings. The study adhered to the Declaration of Helsinki, received ethics approval, and participation was voluntary. Responses were analyzed dichotomously (correct AF or SR) using generalized estimating equations to account for multiple interpretations per physician and per ECG. Multivariable regression identified electrocardiographic predictors of diagnostic performance. Based on prior studies reporting 70% to 75% accuracy for single-lead ECG (SL-ECG) interpretation,^2^ a sample of approximately 200 participants would provide a 95% CI with a ±6% margin of error. Statistical analyses were performed using IBM SPSS Statistics, version 28.

## Results

A total of 196 PCPs completed the questionnaire; 50% reported monthly exposure to smartwatch ECGs and 39% every few months, with low confidence in 48% and average confidence in 40%. Clinical experience was distributed as >10 years (10%), 5 to 10 years (32%), 2 to 5 years (23%), and ≤2 years (35%). Negative QRS polarity, artifacts, and paced rhythm were present in 35%, 35%, and 10% of AF tracings, respectively, vs 15%, 30%, and 10% of SR tracings; coarse AF was present in 35% of cases. PCPs identified 74.2% of AF and 75.2% of SR tracings, with 13.2% of SR misclassified as AF. For AF, the sensitivity was 74.2% (95% CI: 60.7-87.7) and specificity was 85.0% (95% CI: 75.8-94.1). The positive predictive value (PPV) and negative predictive value were 83.2% and 75.4%, respectively. Predictors of incorrect AF interpretation included negative QRS polarity (OR: 4.2; 95% CI: 3.6-4.7), artifacts (OR: 1.5; 95% CI: 1.3-1.6), and paced rhythms (OR: 4.6; 95% CI: 4.0-5.3). Factors that improved accuracy included coarse AF (OR: 0.49; 95% CI: 0.44-0.54) and larger QRS amplitude (OR: 0.91; 95% CI: 0.90-0.94). Frequent exposure to SL-ECGs was protective (OR: 0.45; 95% CI: 0.30-0.66), whereas clinical experience and confidence were not. Erroneous automated interpretations strongly predicted misclassification (OR: 32.3; 95% CI: 21.1-49.4). For SR, errors were more likely with artifacts (OR: 3.5; 95% CI: 3.1-4.0), premature beats (OR: 5.9; 95% CI: 5.1-6.7), and negative QRS polarity (OR: 2.08; 95% CI: 1.8-2.4). False automated interpretations similarly increased SR misclassification (OR: 16.5; 95% CI: 13.1-20.7).

## Discussion

**Figure 1:**
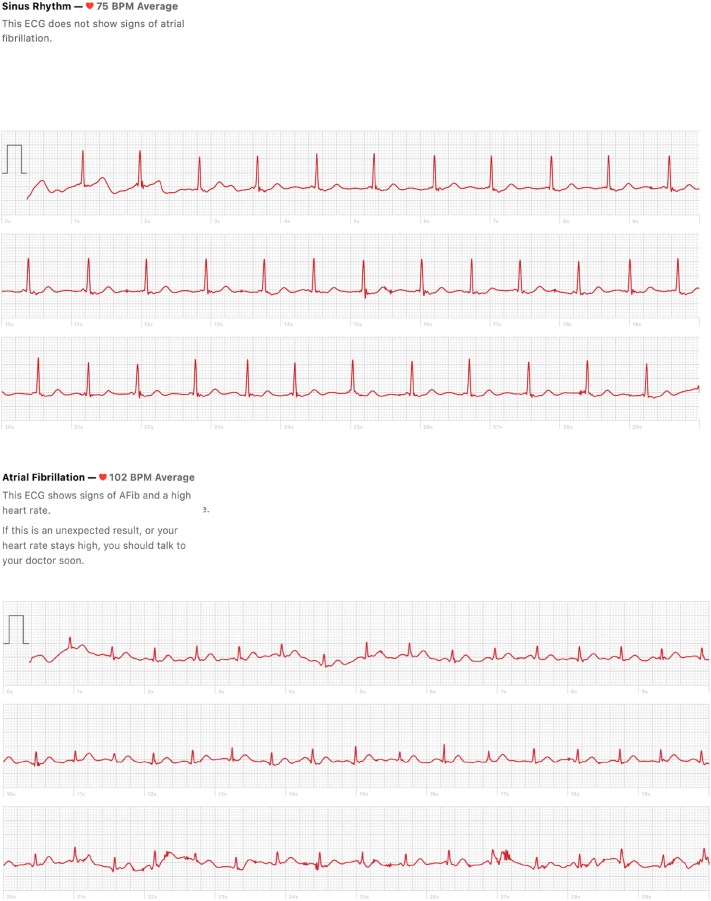
Examples of AF and False AF Smartwatch ECGs Upper panel: Single-lead smartwatch electrocardiogram showing atrial fibrillation with an incorrect automated interpretation. The rhythm is irregularly irregular, and distinct P waves are absent. Lower panel: A tracing of sinus rhythm with baseline artifacts, falsely interpreted by the algorithm as atrial fibrillation. AFib = atrial fibrillation; BPM = beats per minute; ECG = electrocardiogram.

Our findings underscore the challenges of integrating smartwatch-based SL-ECGs into real-world practice. PCPs showed moderate accuracy in AF interpretation (74.2% sensitivity, 85.0% specificity), with misclassification associated with QRS polarity, artifacts, pacing, and automated interpretation. Currently, PCPs appear insufficiently equipped to interpret these recordings reliably. The assumption that proficiency with standard 12-lead ECGs translates to accurate interpretation of smartwatch-derived SL-ECGs is largely unsubstantiated, as conventional ECG expertise does not necessarily extend to SL-ECG analysis.^2^ Moreover, electrocardiographic factors recognized decades ago, namely low amplitude, artifacts, premature beats, and intermittent pacing, remain relevant despite this technological advance. Empirical data on PCPs’ diagnostic efficacy with SL-ECGs remain sparse. PCPs interpreting data from a smartphone-based ECG device showed high sensitivity but low PPV (45.7%) with frequent AF misclassification.^3^ In our study, PCPs achieved only moderate accuracy in interpreting AF and SR on smartwatch SL-ECGs, and about 1 in 4 AF cases were actually missed. The relatively decent PPV observed is influenced by the artificially high prevalence of AF in the study design. In fact, several concerns among PCPs appear to hinder the clinical adoption of smartwatches, including time constraints, limited reimbursement, and skepticism about their accuracy.^4^ Notably, as shown in Figure 1, erroneous automated interpretations had a strong influence on PCPs’, introducing algorithm-induced bias. This aligns with data from conventional ECG machines, where incorrect interpretations have been shown to reduce reader accuracy and increase reliance on algorithmic outputs,^5^ particularly among less-experienced physicians. Overreliance on smartwatch labeling could result in inappropriate anticoagulation, missed AF diagnoses, and delayed intervention, emphasizing the need for caution in clinical decision-making.

In light of these concerns and the expanding role of smartwatch technology in telecardiology, proactive strategies are needed. Professional societies should clearly define which clinicians are qualified to interpret SL-ECGs and implement standardized protocols for ambiguous tracings, particularly in high-risk patients (eg, prior stroke or elevated CHA_2_DS_2_-VASc score). Interim solutions could include online cardiology consultations, though many arrhythmias may still be indistinguishable on SL-ECGs, even to expert readers. Traditional medical curricula must be modernized to integrate SL-ECG training, reflecting the evolving digital health landscape. Emerging artificial intelligence–assisted ECG interpretation and decision support tools offer significant potential to enhance diagnostic accuracy. These systems can help clinicians safely integrate smartwatch ECG data into clinical workflows while leveraging large data sets to detect subtle patterns that may be missed by human interpretation, thereby complementing telecardiology support.

Limitations include potential selection bias due to recruitment process, which may preferentially attract more confident or tech-savvy PCPs, and measurement bias from author-selected ECGs. Tracings were interpreted in a study setting, not within routine clinical workflow. The use of a balanced data set of AF and SR recordings does not reflect clinical prevalence and may introduce spectrum bias. Generalizability is further limited by the inclusion of Apple Watch tracings only, and the relatively small sample size.

In conclusion, PCPs demonstrated suboptimal accuracy in interpreting AF on smartwatch ECGs. As these devices are increasingly integrated into clinical practice, targeted training is required to improve interpretation of consumer-grade ECGs and mitigate overreliance on automated device classifications.

## Funding support and author disclosures

The authors have reported that they have no relationships relevant to the contents of this paper to disclose.
